# Impaired erythropoietin synthesis in chronic kidney disease is caused by alterations in extracellular matrix composition

**DOI:** 10.1111/jcmm.13319

**Published:** 2017-08-30

**Authors:** Gemma Olmos, José M. Muñoz‐Félix, Inés Mora, Anton Gerhard Müller, Maria Piedad Ruiz‐Torres, José M. López‐Novoa, Diego Rodríguez‐Puyol

**Affiliations:** ^1^ Department of System Biology Universidad de Alcalá Alcalá de Henares Madrid Spain; ^2^ REDinREN (Instituto de Salud Carlos III) Madrid Spain; ^3^ IRSIN Instituto Reina Sofía de Investigaciones Nefrológicas Madrid Spain; ^4^ Department of Physiology and Pharmacology Universidad de Salamanca Salamanca Spain; ^5^ Centre for Tumour Biology Barts Cancer Institute Queen Mary University of London London UK; ^6^ Department of Nephrology and Rheumatology University Medical Center Goettingen University Goettingen Goettingen Germany; ^7^ Instituto de Investigaciones Biomédicas de Salamanca (IBSAL) Salamanca Spain; ^8^ Research Unit and Nephrology Section Hospital Príncipe de Asturias and Department of Medicine Universidad de Alcalá Alcalá de Henares Madrid Spain

**Keywords:** anaemia, fibrosis, erythropoietin, chronic kidney disease, hypoxia‐inducible factor

## Abstract

Renal fibrosis and anaemia are two of the most relevant events in chronic kidney disease. Fibrosis is characterized by the accumulation of extracellular matrix proteins in the glomeruli and tubular interstitium. Anaemia is the consequence of a decrease in erythropoietin production in fibrotic kidneys. This work analyses the possibility that the accumulation of abnormal collagens in kidney interstitium could be one of the mechanisms responsible for erythropoietin decreased synthesis. In renal interstitial fibroblast grown on collagen I, erythropoietin mRNA expression and HIF‐2α protein decreased, whereas focal adhesion kinase protein (FAK) phosphorylation and proteasome activity increased, compared to cells grown on collagen IV. Proteasome inhibition or FAK inactivation in cells plated on collagen I restored erythropoietin and HIF‐2α expression. FAK inhibition also decreased the collagen I‐dependent proteasome activation. In a model of tubulointerstitial fibrosis induced by unilateral ureteral obstruction in mice, increased collagen I protein content and an almost complete disappearance of erythropoietin mRNA expression were observed in the ureteral ligated kidney with respect to the contralateral control. Interestingly, erythropoietin synthesis was recovered in obstructed mice treated with proteasome inhibitor. These data suggest that reduced kidney erythropoietin synthesis could be caused by the accumulation of abnormal extracellular matrix proteins.

## Introduction

Extracellular matrix (ECM) is one of the main components of the different tissues and organs. It plays a critical role in maintaining the structure of tissues, but it also modulates the phenotype of adjacent cells. ECM is composed by proteins such as laminin, fibronectin and collagens. In pathophysiological conditions, ECM proteins accumulate in the tissue, and abnormal ECM components appear in organs in which they are not usually present 1. ECM interacts with cells through different surface transmembrane receptors, including integrins. Integrins are a family of heterodimeric receptors composed by two non‐covalently associated subunits, α and β, which mediate signalling from ECM to the cellular cytoplasm and generate intracellular signals regulating multiple actions such as proliferation and survival 2. The major signalling pathways are launched by the activation of the well‐known integrin mediators, integrin‐linked kinase (ILK) 3 or focal adhesion kinase (FAK), which causes phosphorylation of other intracellular proteins 4, 5, activating routes as MAPK 6, PI3/AKT 7 or RhoB 8.

In chronic kidney disease (CKD), there is an unusual accumulation of ECM proteins in the renal interstitium, a finding known as interstitial fibrosis. In this process, one of the major fibrillar collagens, collagen type I (COLI), accumulates in the interstitium, whereas the relative amount of collagen type IV (COLIV) decreases 9. These changes in ECM provoke important modifications in the renal structure, as well as in the function of tubule‐interstitial cells, with the progressive loss of renal function 10, 11, 12, 13. The main mechanism responsible for excessive pathological extracellular matrix deposition seems to be the proliferation and activation of interstitial fibroblast to myofibroblasts and the subsequent increase in ECM synthesis and release 14, 15, 16.

One of the most relevant clinical events in CKD is the anaemia produced by a decreased erythropoietin (EPO) production 17. In adults, EPO is mainly synthesized in the kidney and it is necessary for effective haematopoiesis. Its production is dependent on oxygen concentration 18. In the last years, several studies have tried to identify EPO‐producing cells in kidney. A number of evidence support that EPO is synthesized in interstitial fibroblast‐like cells in the deep renal cortex and in the outer renal medulla 19, 20, 21, 22, 23. EPO synthesis is regulated by the transcription factor, hypoxia‐inducible factor, HIF 24. HIF is a heterodimeric basic helix–loop–helix formed by α subunit regulated by oxygen levels (isoforms HIF1α and HIF2α) and a constitutively expressed β subunit. In the kidney, they are expressed in tubular epithelial, endothelial, glomerular and interstitial cells 25, 26, 27. A number of important evidence supports that HIF2α is the main responsible for the synthesis and regulation of EPO 28, 29, 30, 31, 32, 33. In normoxia conditions, HIFα is hydroxylated by specific iron‐dependent dioxygenase enzymes called prolyl hydroxylases (PHD) 34. These enzymes hydroxylate HIFα at specific proline residues, which are recognized by a tumour suppressor protein, the von Hippel–Lindau protein (pVHL), and ubiquitined. These post‐translational modifications target HIFα for degradation by proteasome 35, 36, 37, 38, 39. Under hypoxia, HIFα is not hydroxylated but accumulates and it is bound to HIFβ. This complex acts as a transcription factor inducing transcription of genes involved in different processes such as angiogenesis, apoptosis or metabolism 40.

CKD‐related anaemia is usually considered the result of the progressive loss of EPO‐producing interstitial cells. However, taking into consideration the relevance of ECM proteins in the modulation of cell phenotype, it may also be suggested that the interstitial fibrosis that takes place in CKD could also be responsible for the changes in EPO synthesis. Few studies have been devoted to establishing a link between ECM proteins and EPO synthesis. A recent study has shown that, in renal fibrosis, EPO‐producing cells possess plasticity and their transition to myofibroblasts can be the link between anaemia and renal fibrosis in CKD 41. However, the mechanism responsible for these relationships is still unclear. To understand the mechanisms that may link erythropoietin decreased synthesis and renal fibrosis, we used an *in vitro* approach using human renal interstitial fibroblast cultured on different collagen matrix, and an *in vivo* model of tubulointerstitial fibrosis induced by unilateral ureteral ligation (UUO) in mice. Our results suggest that the accumulation of abnormal collagens may be one of the mechanisms involved in the decreased EPO synthesis. In addition, they show that EPO changes are due to proteasome‐dependent HIF2α protein degradation, induced by FAK activation.

## Materials and methods

### Materials

Foetal calf serum (FCS), foetal bovine serum (FBS), lipofectamine, antibiotics, Optimen, RNA*later* solution, High‐Capacity cDNA Reverse Transcription Kit gene expression assays and anti‐GAPDH mouse antibody were from Life Technologies (Carlsbad, CA, USA). Cell culture medium DMEM was from Lonza (Basel, Switzerland). Collagen I antibody was from Chemicon International Inc (Ternecula, CA, USA). Antibodies against phosphorylated form of FAK and GSK and against ILK and total‐Fak were from Cell Signalling Technology (Danvers, MA, USA). Anti‐HIF‐2α and anti‐erythropoietin antibodies were from Abcam (Cambridge, UK). Anti‐OH‐HIF antibody was from Novus Biologicals (Littleton, CO, USA). Anti‐rabbit antibody was from Dako (Barcelona, Spain). Anti‐rabbit Cy3 was from Jackson Immunoresearch (West Grove, PA, USA), and antimouse 488 Alexa and Hoechst were from Molecular Probes (Barcelona, Spain). Dual‐Luciferase Reporter Assay System was from Promega (Madison, WI, USA). FAK inhibitor 4‐amino‐5‐(4‐chloro‐phenyl)‐7‐(*t*‐butyl)pyrazolo3,4‐dpyrimidine (PP2), Fluorogenic substrate Succ‐LLVY‐AMC for proteasome activity and proteasome inhibitor MG132 were purchased from Calbiochem (San Diego, CA, USA). Proteasome inhibitor bortezomib (Velcade^®^) was from Janssen‐Cilag International (Beerse, Belgium). siRNA negative control and siFAK were from Santa Cruz Biotechnology (Santa Cruz, CA, USA). Prolong antifade was from Invitrogen (Barcelona, Spain). Chemiluminescence reagent was from Thermo Scientific (Basingstoke, UK). FastStart DNA master SYBR Green I Kit was from Roche (Mannheim, Germany). Other reagents and antibodies were from Sigma Chemical (St. Louis, MO, USA).

### Primers

The following primers were used: EPO human, 5′‐TCTATGCCTGGAAGAGGATGGAGGTCG‐3′ (forward) and 5‐TGCGGAAAGTGTCAGCAGTGATTGTTC‐3′ (reverse); HIF‐2α human 5′‐AGGACTACAGCCTGTCGTCAGC‐3′ (forward) and 5′‐GAGGTGCGAGGACGTTCC‐3′(reverse); PHD2 human 5′‐GCACGACACCGGGAAGTT–3′ (forward) and 5′‐CCAGCTTCCCGTTACAGT‐3′ (reverse); GAPDH human 5′‐TCCACTGGCGTCTTCACC‐3′(forward) and 5′‐GGCAGAGATGATGACCCTTTT‐3 (reverse); EPO mouse 5′ CATCTGCGACAGTCGAGTTCTG‐3′(forward) and 5′‐CACAACCCATCGTGACATTTTC‐3′(reverse); GAPDH mouse 5′‐GTCGGTGTGAACGGATTTG–3′ (forward) and 5′‐GAATTTGCCGTGAGTGGAGT‐3′ (reverse).

### Cell culture and animal studies

Renal interstitial fibroblasts TK173 42 and HepG2 cells (from American Type Culture Collection; Rockville, MD, USA) were maintained in culture medium, and plates were coated with COLI or COLIV following previous descriptions 43. For experiments, cells were grown for the indicated times in serum‐deprived DMEM. For hypoxia conditions, cells were incubated in one hypoxia workstation (Ruskinn Invivo200) at 1% O_2_, 5%CO_2_ and 94% N_2_ for 24 hrs. Unilateral ureteral obstruction (UUO) was performed in C57BL/6 mice as previously described in 44. Ligation was maintained for 15 days until the mice were killed, and 13 days after obstruction, some animals received bortezomib (750 μg/Kg i.p., in isotonic saline), an inhibitor of proteasome activity, or isotonic saline. In all procedures, mice were treated in accordance with the Recommendations of the Helsinki Declaration on the Advice on Care and Use of Animals referred to in: law 14\/2 007 (3 July) on Biomedical Research, Conseil de l′Europe (published in Official Daily N. L358/1‐358/6, 18‐12‐1986), Government Spanish (Royal Decree 223/1 988, (14 March) and Order of 13‐10‐1989, and Official Bulletin of the State b. 256, pp. 31349‐31362, 28‐10‐1990). The procedure was approved for the Bioethics committee of the University of Salamanca.

### Renal tissue preparation

Obstructed and non‐obstructed kidneys were recovered 15 days after surgery and perfused with heparinized saline solution. Next, kidneys were halved longitudinally in order to use one half for protein and RNA extraction and the other half for histological studies. Renal samples for protein and mRNA extraction were frozen in liquid nitrogen and stored at −80°C and for histological studies were fixed in formaldehyde for 24 hrs and then embedded in paraffin.

### Histochemistry and double‐immunofluorescence

3 μm sections were cut from paraffin‐embedded samples and stained with Masson's trichrome and Sirius red staining 44. Sirius red staining was evaluated by a quantitative scoring system, Image‐Pro Plus software (Media Cybernetics, Bethesda, MD, USA) in five randomly selected fields per kidney (×200). For double‐immunohistochemistry, heat‐induced antigen unmasking was performed in Tris 10 mM–EDTA 1 mM, pH 8.00 and washed with PBS. Sections were incubated simultaneously with anti‐α‐SMA and anti‐HIF2α antibodies for 1 hr at room temperature. Following three washes in PBS, sections were incubated with anti‐rabbit Cy3 and antimouse 488 Alexa, diluted 1:1000 for 45 min. at room temperature, washed in PBS and stained with 2 mM Hoechst. Slides were rinsed in PBS and mounted in Prolong antifade (Invitrogen, Barcelona, Spain). Images were photographed with a Zeiss Axiovert 200M microscope (Barcelona, Spain) and a Zeiss LSM 510 confocal module (Barcelona, Spain).

### Proteasome activity

Cells were plated over COLIV or COLI at indicated times. Then, they were lysed in lysis buffer (50 mM Tris–HCl, pH 7.4, 5 mM MgCl2, 2 mM DTT and 2 mM ATP). Samples were frozen at −80°C and thawed three times and then centrifuged for 10 min. at 10,000 × *g*. Proteasome activity was measured in the supernatant fraction using the fluorogenic substrate (80 μM) Succ‐LLVY‐AMC. Equal fractions of the supernatants were pre‐incubated with the proteasome inhibitor MG132 (10 μM), for 30 min. at 37°C before addition of the substrate. The fluorescence was determined by Luminescence spectrometer LS50 (Perkin Elmer, Wellesley, MA, USA) (380 nm excitation wavelength and 460 nm emission wavelength). Activities of enzymes were measured as arbitrary unit/min/mg protein and then expressed as percentage of the activity found in cells plated on COLIV, used as control cells.

### Western blot

Treated cells were washed with cold PBS, lysed in buffer (50 mM Trizma, pH 8.0, 150 mM NaCl, 0.1% Triton X‐100, 10 mM EDTA, 0.25% sodium deoxycholate and protease inhibitors) and were finally sonicated. Protein samples were run onto 8–12% SDS‐PAGE gels and then were transferred to PVDF membranes, which were blocked with 5% non‐fat dry milk or 3% BSA in TBS‐T (50 mM Trizma, pH 7.6, 150 mM NaCl, 0.1% Tween‐20). Membranes were incubated overnight at 4°C with the corresponding primary antibodies and then incubated with secondary antibodies at room temperature for 1 hr. Immunoblots were detected by chemiluminescence. Densitometry analyses were performed with NIH ImageJ software (http://rsbweb.nih.gov/ij/).

### Quantitative reverse transcription PCR

Total RNA was extracted and quantified, and 2 μg of total RNA was retrotranscribed to cDNA with High Capacity cDNA Reverse Transcription Kit. 250 ng of this cDNA was used as a template for PCR amplification with the LC FastStart DNA master SYBR Green I Kit following the manufacturer's instructions. The normalized gene expression method (2^−ΔΔCT^) for relative quantification of gene expression was used.

### Small‐interfering RNA transfection

To deplete expression of FAK protein, a specific small‐interfering RNA (siRNA) was used. Cells were transfected in 1 ml of OPTIMEM with 200 nM siRNA or Silencer TM negative control (Scrambled RNA) using lipofectamine 2000 for 24 hrs. Then, 1 ml of medium DMEN containing FCS was added for 24 hrs. Afterwards, cells were plated on COLIV or COLI as indicated above.

### Transient transfection and Luciferase assays

Cells were transfected using lipofectamine reagent with 0.1 μg of reporter plasmid p9HIF1‐Luc (kindly provided by Dr. Landazuri, University Autónoma, Madrid) and 0.01 μg of Renilla plasmid luciferase reporter as internal control. Afterwards, the cells were treated and lysed, and their luciferase activity was measured with Dual‐Luciferase Reporter Assay System according to the manufacturer's instructions in a Lumat LB9506 luminometer (Berthold Technologies, Herts, UK). Luciferase activity was normalized based on Renilla luciferase activity and protein content.

### Statistical analysis

Results are expressed as the mean ± standard error of the mean (S.E.M.) of a variable number of independent experiments detailed in Figure Legends. Some experiments are presented as fold increase or percentage of control values. In the *in vitro* experiments, comparisons were performed by the Student's *t*‐test (two groups) or one or two‐way ANOVA (three or more groups, one or two variables). In the *in vivo* experiments, the Mann–Whitney test was used. A *P* < 0.05 was considered statistically significant.

## Results

### Culture on COLI induces EPO downregulation in interstitial fibroblast

**Figure 1:**
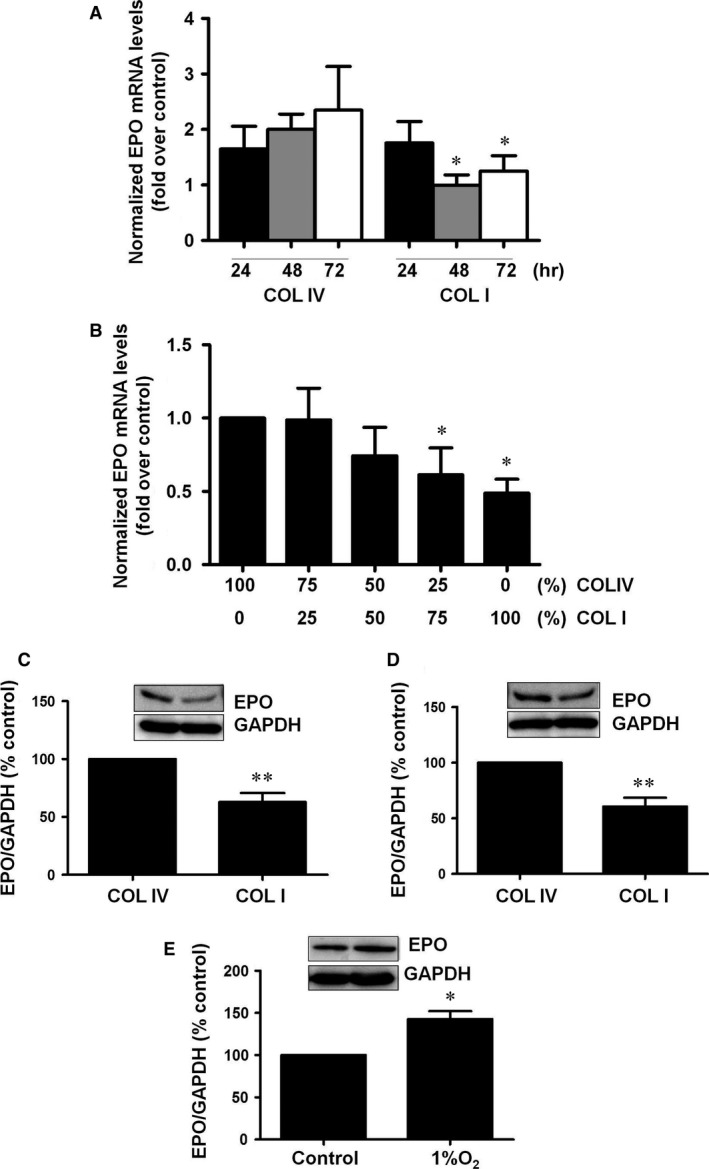
Collagen I down‐regulates erythropoietin mRNA and protein levels in interstitial fibroblast cells. (**A**) Cells were grown on plates collagen IV (COLIV) or collagen I (COLI) for 24, 48 and 72 hrs. Erythropoietin mRNA levels were determined by quantitative RT‐PCR. Relative fold change values were normalized against GAPDH as endogenous control and expressed as fold induction over COLIV. (**B**) Cells were grown on plates coated with different proportions of COLIV/COLI for 48 hrs. Erythropoietin mRNA levels were determined by quantitative RT‐PCR. Relative fold change values were normalized against GAPDH as endogenous control and expressed as fold induction over COLIV. (**C**) TK173 cells and (**D**) HepG2 cells were grown on COLIV or COLI for 48 hrs. EPO protein levels and GAPDH as endogenous control were evaluated by immunoblotting. A representative blot is shown. Bar graphs represent percentage of densitometric levels *versus* COLIV as control. (**E**) TK173 cells were submitted under normoxia or hypoxia (1% O_2_) conditions for 24‐hrs EPO protein levels and GAPDH as endogenous control was evaluated by immunoblotting. A representative blot is shown. Bar graphs represent percentage of densitometric levels *versus* control. Results are mean ± S.E.M.; *N* = 3‐5 independent experiments (*P < 0.05 *versus* COLIV; **P < 0.01).

To analyse the effects of changes in the ECM composition on EPO synthesis, TK173 cells, a line of renal interstitial fibroblast, were used. TK173 cells were grown on COLI or COLIV for variable times. Figure 1A shows that EPO mRNA expression was significantly lower at 48 hrs and at 72 hrs culture on COLI with respect to COLIV, suggesting a time‐dependent regulation of hormone synthesis in the presence of COLI in the culture substrate. The observed changes also depended on the proportion of COLI. In cells plated with different collagen proportions for 48 hrs, we observed a proportion‐dependent decrease in EPO mRNA: when COLI proportion augmented, EPO mRNA decreased (Fig. 1B). Changes in EPO mRNA were confirmed by analysing EPO protein content by Western blot. We observed a significant decrease in EPO protein in cells cultured on COLI compared to COLIV for 48 hrs (Fig. 1C). Similar COLI‐dependent changes in EPO protein content were observed in other well‐known EPO‐producing cells, HepG2 (Fig. 1D). Hypoxia, the physiological stimulus of EPO synthesis, increased EPO protein levels in TK173 cells after 24 hrs (Fig. 1E). Taken together, these results indicate that erythropoietin is down‐regulated in a time‐ and proportion‐dependent manner when cells are grown on COLI *versus* COLIV.

### Culture on COLI induces HIF2α degradation

**Figure 2:**
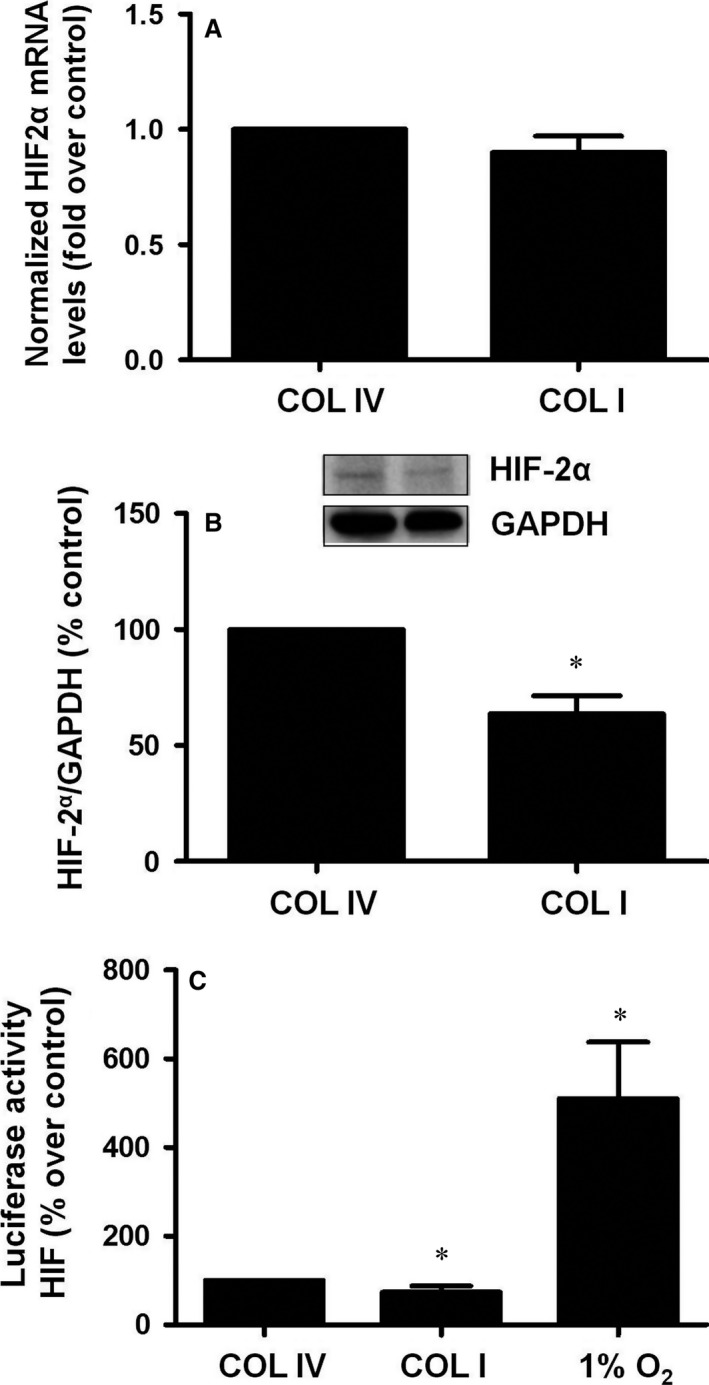
Collagen I down‐regulates HIFα protein and activity levels in interstitial fibroblast cells. Cells were grown on collagen IV (COLIV) or collagen I (COLI) for 48 hrs. (**A**) HIF2α mRNA levels were determined by quantitative RT‐PCR. Relative fold change values were normalized against GAPDH as endogenous control and expressed as fold induction over COL IV as control. (**B**) HIF2α protein levels and GAPDH as endogenous control were evaluated by immunoblotting. A representative blot is shown. Bar graphs represent percentage of densitometric levels *versus* COLIV as control. (**C**) Luciferase activity was determined in lysates of cells transfected with a reporter as indicated in methods. Each bar represents the luciferase activity normalized by the Renilla luciferase activity and expressed as arbitrary units. Results are mean ± S.E.M.; *N* = 3 independent experiments (*P < 0.05 *versus* COLIV).

HIF2α is the main transcription factor involved in the regulation of EPO synthesis in kidney. To analyse its implication in the above‐mentioned EPO synthesis dependency on COLI presence, we analysed mRNA and HIF2α protein levels, as well as HIF activity, in cells cultured on COLI or COLIV. Figure 2A shows no differences in HIF2α mRNA levels In contrast, we observed a significant decrease in HIF2α protein levels in cells grown on COLI compared to COLIV cultured cells (Fig. 2B). To determine HIF activity, we used a hypoxia‐responsive element (HRE)‐driven reporter construct. A luciferase plasmid that contained nine copies of the human HIF binding sequence located between positions −985 and −951 of the 5′‐human vascular endothelial growth factor gene promoter 45 was transiently transfected into cells. In this assay, cells cultured on COLI showed a significantly lower luciferase HIF activity compared to COLIV and significantly increased when cells were under hypoxia conditions for 24 hrs (Fig. 2C). These results demonstrate that HIF2α protein decreases in cells cultured on COLI by a mechanism independent of mRNA expression and probably dependent on HIF2α degradation.

### Culture on COLI does not affect the prolyl hydroxylase‐2 activation but increases proteasome activity

**Figure 3:**
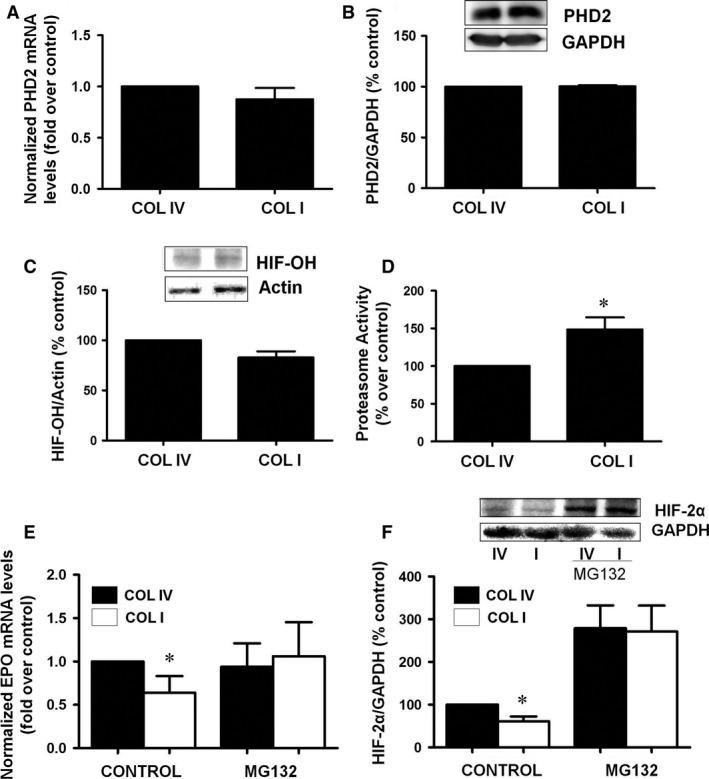
Collagen type I does not affect prolyl hydroxylases activity, increases proteasome activity and proteasome inhibition restores erythropoietin levels. Cells were grown on collagen IV (COLIV) or collagen I (COLI) for 48 hrs. (**A**) PHD2 mRNA levels were determined by quantitative RT‐PCR. Relative fold change values were normalized against GAPDH as endogenous control and expressed as fold induction over COLIV as control. (**B**) PHD2 protein levels and GAPDH as endogenous control were evaluated by immunoblotting. A representative blot is shown. (**C**) Cells were grown on COLIV or COLI for 24 hrs and then treated with proteasome inhibitor MG132 for another 24 hrs. Hydroxylated‐HIF1α protein levels and actin as endogenous control were evaluated by immunoblotting. A representative blot is shown. (**D**) Cells were grown on collagen IV (COLIV) or collagen I (COLI) for 48 hrs. Proteasome activity was measured as fluorogenic chymotrypsin substrate in a fluorimeter. Data are shown as the percentage of activity measured on COLIV. Cells were grown on COL IV or COLI for 24 hrs and then treated with proteasome inhibitor MG132 for another 24 hrs. (**E**) Erythropoietin mRNA levels were determined by quantitative RT‐PCR. Relative fold change values were normalized against GAPDH as endogenous control and expressed as fold induction over COLIV. Data are shown as the percentage of activity measured on COLIV. (**F**) HIF2α protein levels and GAPDH as endogenous control were evaluated by immunoblotting. A representative blot is shown. Bar graphs represent percentage of densitometric levels *versus* COLIV as control. Results are mean ± S.E.M.; *N* = 3 independent experiments (*P < 0.05 *versus* the other groups).

As PHD enzymes regulate HIF degradation, we analysed the expression of these enzymes in human fibroblasts. We found that PHD2 was the predominant form detected in these cells. Neither its mRNA expression (Fig. 3A) nor its protein content (Fig. 3B) was modified by the two collagens used as culture substrate. To ensure that HIF2α degradation was not dependent on a PHD increased activity, we studied HIF hydroxylation with a specific antibody that recognizes HIF hydroxylated in the presence of the proteasome inhibitor MG132 to avoid hydroxylated protein degradation. We did not observe any increment of HIF hydroxylation in cells grown on COLI with respect to COLIV grown cells (Fig. 3C). Thereafter, to study the relevance of the main pathway involved in HIF degradation, we measured proteasome activity in cells cultured in both types of collagens tested. Cells cultured on COLI showed a higher proteasome activity than those cultured on COLIV (Fig. 3D). When proteasome activity was inhibited by MG132, EPO mRNA expression (Fig. 3E) and HIF‐2α protein level (Fig. 3F) were similar in COLI than in COLIV grown cells. These results support the premise that COLI effects on EPO synthesis are mediated by the direct induction of proteasome activity.

### COLI‐dependent HIF2α‐EPO downregulation and increased proteasome activity are mediated by FAK

**Figure 4:**
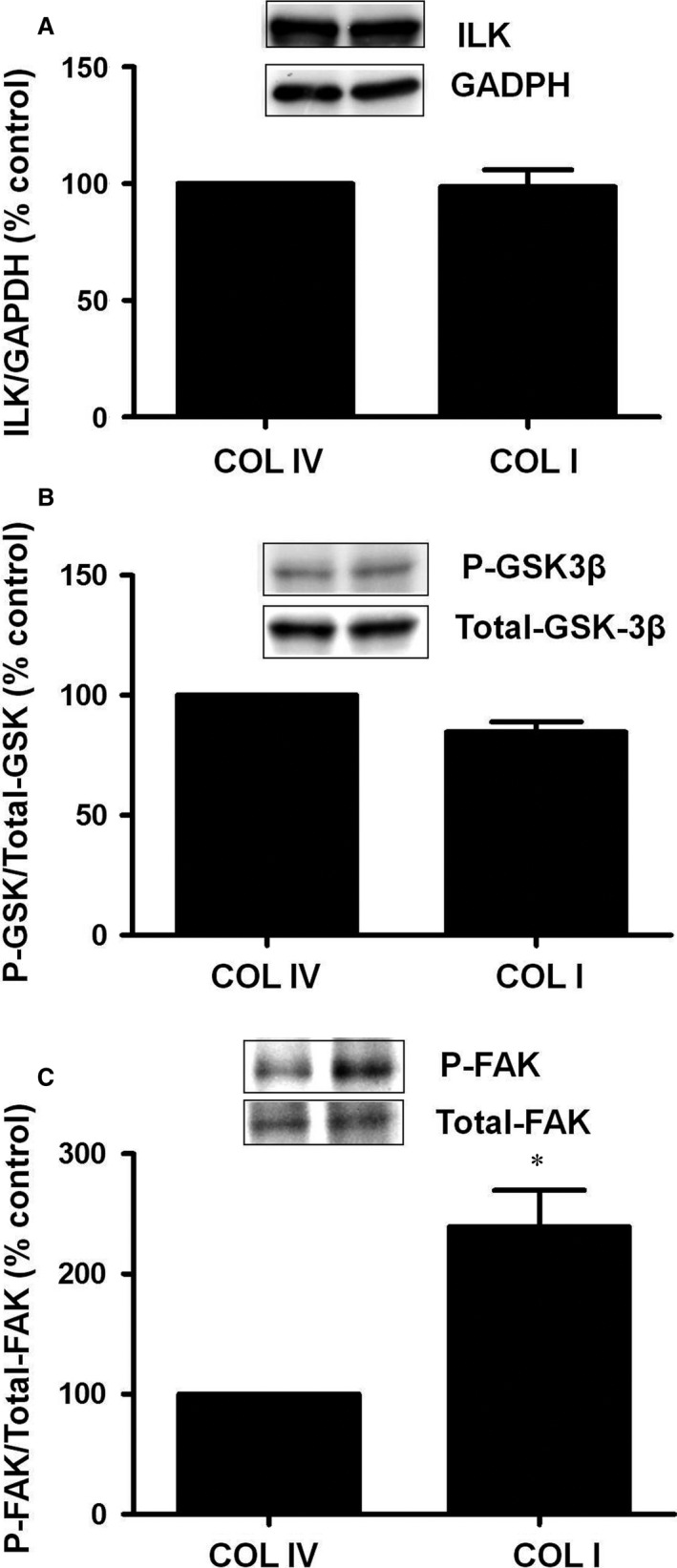
Collagen I activates focal adhesion kinase (FAK) but not ILK. Cells were grown on collagen IV (COLIV) or collagen I (COLI) for 48 hrs. (**A**) ILK protein and GAPDH as endogenous control, (**B**) Phospho‐GSK protein and Total‐GSK as endogenous control and (**C**) Phospho‐FAK protein and Total‐FAK as endogenous control were evaluated by immunoblotting. A representative blot of each one is shown. Bar graphs represent percentage of densitometric levels *versus* COL IV. Results are mean ± S.E.M.; *N* = 3 independent experiments (*P < 0.05 *versus* COLIV).

**Figure 5:**
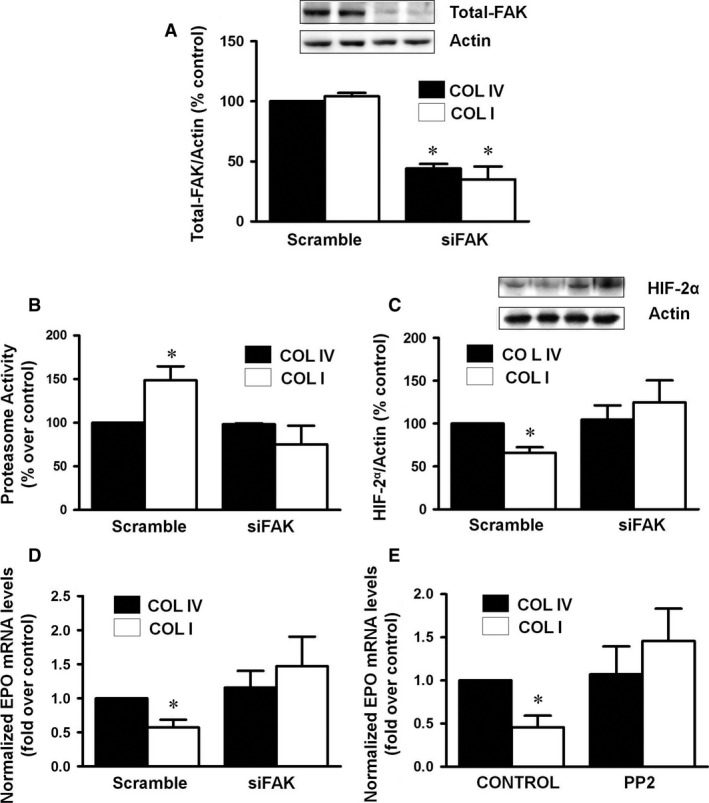
Deleted FAK restored proteasome activity at basal levels, inhibits HIF‐2α degradation and restores erythropoietin levels. Cells were depleted of FAK with a specific small‐interfering RNA (siFAK), and a scrambled RNA (Scramble) was used as control. (**A**) Expression of Total‐FAK protein was analysed by Western blot. Equal protein loading was confirmed by probing with actin as endogenous control. A representative Western blot is shown. Cells transfected with siRNA against FAK protein or scramble were grown on collagen IV (COLIV) or collagen I (COLI) for 48 hrs. Then, it was determined: (**B**) Proteasome activity measured as fluorogenic chymotrypsin substrate in a fluorimeter. Data are shown as the percentage of activity measured on COLIV. (**C**) HIF2α protein levels and actin as endogenous control were evaluated by immunoblotting. A representative blot is shown. (**D**) Erythropoietin mRNA levels were determined by quantitative RT‐PCR. Relative fold change values were normalized against GAPDH as endogenous control and expressed as fold induction over COLIV. (**E**) Cells were grown on collagen IV (COLIV) or collagen I (COLI) for 24 hrs and then incubated with the FAK inhibitor 4‐amino‐5‐(4‐chloro‐phenyl)‐7‐(*t*‐butyl) pyrazolo3,4‐dpyrimidine (PP2) 10 μM for other 24 hrs. Erythropoietin mRNA levels were determined by quantitative RT‐PCR. Relative fold change values were normalized against GAPDH as endogenous control and expressed as fold induction over COLIV. Results are mean ± S.E.M.; *N* = 3 independent experiments (*P < 0.05 *versus* the other groups).

Collagens are ligands of integrins whose interaction produces a conformational change that initiates signalling cascade involving the integrin mediators ILK and FAK. First, we analysed ILK protein content and activity in cells plated on COLIV or COLI for 48 hrs. Our data show that no changes were observed in ILK protein content and activity measured as GSK3β protein phosphorylation on either of the collagens tested (Fig. 4A and B, respectively). Otherwise, we analysed FAK activation. Cells incubated on COLI exhibited a sustained increase in FAK tyrosine phosphorylation compared to cells plated on COLIV (Fig. 4C). To establish the relevance of COLI‐dependent FAK activation in the previously described changes observed in COL I grown cells, FAK was deleted in cells with a specific siRNA (siFAK), which almost completely abrogated FAK expression (Fig. 5A). In absence of FAK, proteasome activity (Fig. 5B), HIF2α protein content (Fig. 5C) and EPO mRNA expression were similar in COLI than in COLIV grown cells (Fig. 5D). PP2, a selective inhibitor of FAK activation, exerted the same effects as siFAK on the COLI‐induced EPO mRNA downregulation (Fig. 5E). These results show that FAK is a relevant integrin‐linked protein involved in the EPO modulation induced by COLI.

### EPO synthesis decreases after unilateral ureteral obstruction (UUO) and is recovered after proteasome inhibition

**Figure 6:**
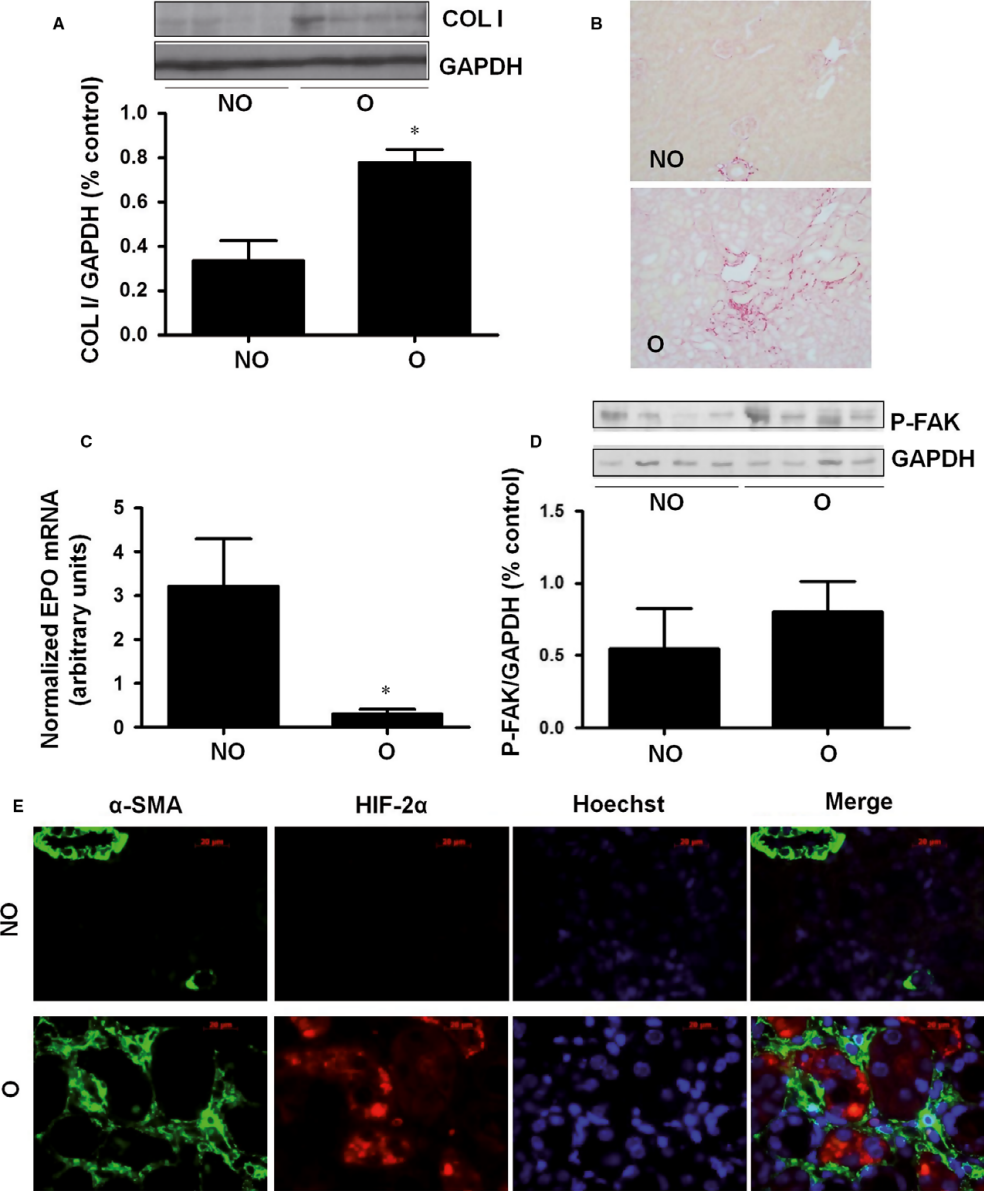
EPO decreased in kidneys with tubulointerstitial fibrosis induced by unilateral ureteral obstruction (UUO). (**A**) Collagen I protein levels and GAPDH as endogenous control were evaluated by immunoblotting in non‐obstructed (NO) and obstructed (O) kidneys. A representative blot is shown. Bar graphs represent percentage of densitometric levels**.** (**B**) Representative images of the renal cortex stained with Sirius red of non‐obstructed (NO) and obstructed (O) kidneys. Bar = 100 μm. (**C**) Erythropoietin mRNA levels from renal cortex were determined by quantitative RT‐PCR. Relative fold change values were normalized against GAPDH as endogenous control and expressed as fold changes over non‐obstructed (NO) kidney. (**D**) Phosphorylated‐FAK protein levels and GAPDH as endogenous control were evaluated by immunoblotting. A representative blot is shown. Bar graphs represent percentage of densitometric levels. (**E**) Representative images of the renal cortex stained with specific antibodies corresponding to non‐obstructed (NO) and obstructed (O) kidneys: αSMA (Green); HIF2α (red), nucleus (blue). Bar = 20 μm. Data are means ± S.E. of five to seven animals/group.*P < 0.05 *versus* non‐obstructed kidney.

**Figure 7:**
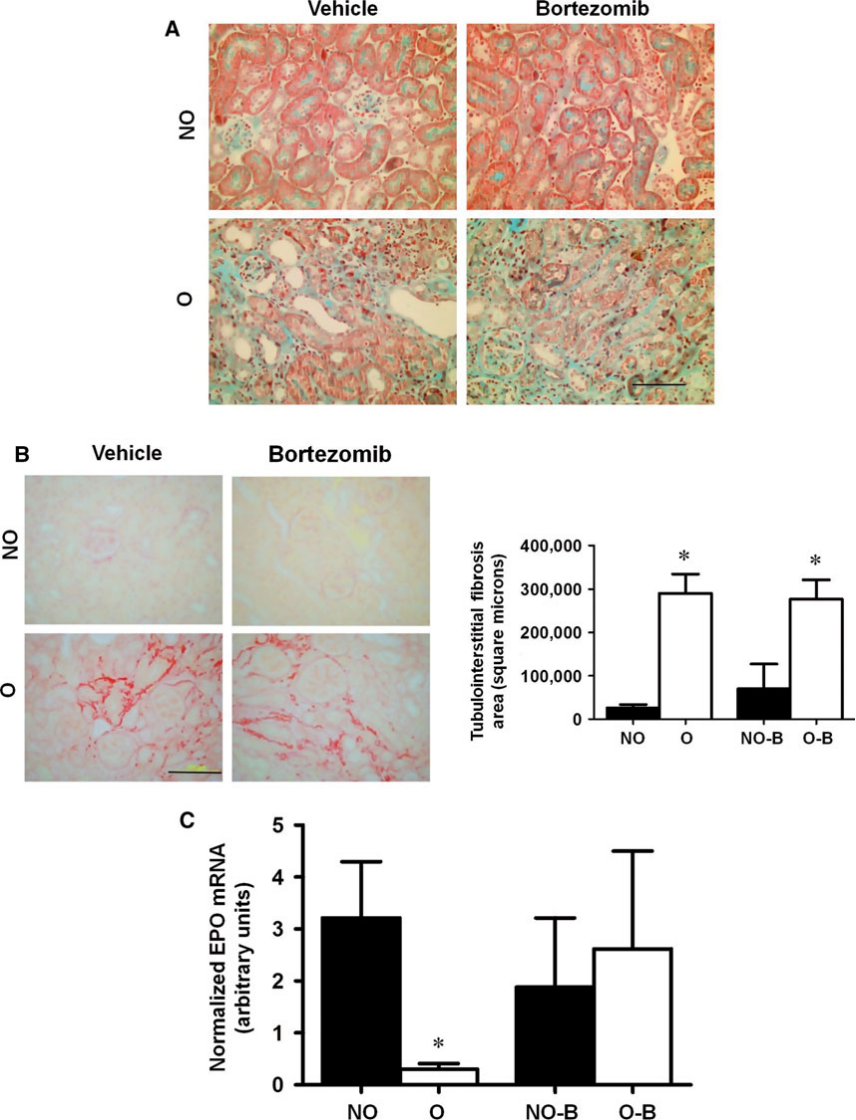
EPO mRNA is recovered in obstructed kidneys of mice with UUO treated with proteasome inhibitor bortezomib. (**A**) Representative images of the renal cortex stained with (**A**) Masson's trichrome and (**B**) Sirius red and quantification by Image‐Pro Plus expressed as square microns corresponding to non‐obstructed (NO), obstructed (O), non‐obstructed treated with bortezomib (NO‐B) or obstructed treated with bortezomib (O‐B) kidneys. Bar = 100 μm. (**C**) Erythropoietin mRNA levels from renal cortex from NO, O, NO‐B and O‐B kidneys were determined by quantitative RT‐PCR. Relative fold change values were normalized against GAPDH as endogenous control and expressed as fold changes over non‐obstructed (NO) kidney. Data are means ± S.E. of five to seven animals/group. *P < 0.05 *versus* non‐obstructed kidney.

The UUO model of tubulointerstitial fibrosis is characterized by the abnormal accumulation of ECM proteins in the obstructed kidney compared to the contralateral non‐obstructed organ. 15 days after surgery, we observed an increased COLI content (Fig. 6A) and fibrosis (Fig. 6B) in obstructed kidneys (O) compared to non‐obstructed (NO) control organs, in agreement with previous results 44. In these experimental conditions, EPO mRNA levels in renal cortex almost completely disappeared in O kidneys (Fig. 6C). With respect to FAK activation, an increased tyrosine phosphorylation of the protein was detected by Western blot in renal cortex from kidneys, but the differences with the NO kidney were not statistically significant (Fig. 6D). The basal HIF2α content in NO kidneys was almost undetectable by immunohistochemistry. Surprisingly, O kidneys showed marked overexpression of the protein in renal epithelial tubular cells but not in interstitial fibroblast, as demonstrated by the lack of co‐localization of HIF2α with the fibroblast marker α‐SMA (Fig. 6E). Furthermore, obstructed kidneys from UUO mice treated with the proteasome inhibitor, bortezomib, showed similar levels of extracellular matrix than O kidneys from mice non‐treated with bortezomib (Fig. 7A and B). In this context of proteasome inhibition and also similar levels of extracellular matrix levels, EPO mRNA levels were higher than the O kidneys from non‐treated mice (Fig. 7C).

## Discussion

One of the main clinical problems in patients with CKD is anaemia. Different factors are responsible for the decreased erythrocyte number in renal patients, being the most relevant in most cases a reduced EPO synthesis. In fact, treatment with human recombinant EPO (rhEPO) significantly improves the anaemia of these patients. Other factors implicated in renal anaemia are blood loss, shortening of red blood cell lifespan, malnutrition, reduced iron availability, and inhibition of the growth of erythrocytic progenitors by inflammatory cytokines and uraemic toxins. The mechanism by which EPO synthesis deficiency develops in CKD subjects has been attributed to a decrease in the number of kidney EPO‐producing cells. EPO seems to be synthesized by a particular subtype of interstitial fibroblast and the loss of these cells, in the progressive interstitial fibrosis that characterizes CKD, has been supposed to be the cause of EPO deficiency 46. However, alternative mechanisms, like phenotypic changes of Epo‐producing renal fibroblasts, that trans‐differentiate to scar‐producing myofibroblasts 47, could be proposed to explain the reduced EPO synthesis. Fibroblast phenotypic changes could be the consequence of the same noxious stimuli that damage kidney structures, but they could also be explained by the abnormal ECM protein accumulation in the renal interstitium.

Renal fibroblasts are a heterogeneous cellular population which have several functions as extracellular matrix production 48, vascular integrity support 49 and EPO synthesis 50. It has not still been possible to separate and cultivate these different types of cells for a better study and thus, many studies about EPO synthesis regulation have been conducted with EPO‐producing hepatoma cells. However, this hepatoma cell model does not appear to be the most adequate to study CKD‐dependent EPO synthesis deficiency, particularly because the mechanism of EPO regulation in liver is different than in kidney 51. Taken into account this fact, the present work was conducted in interstitial fibroblast TK173 as a model closer to the kidney. Under the experimental conditions selected, EPO synthesis by TK173 cells appears comparable to that of hepatome‐derived cells, and its expression depends on the O_2_ partial pressure, as observed in the experiments under hypoxic conditions.

We observed that the interstitial renal fibroblast cultured on plates coated with COLI, a fibrillar collagen that is not usually present in the renal interstitium but that appears under pathophysiological conditions, exhibited a time‐dependent decrease in EPO mRNA levels and protein content compared to cells grown on COL IV, considered the physiological collagen component of renal ECM, as it is a major component of epithelial basal membranes 9. As interstitial fibrosis could be better mimicked by a variable mix of different ECM proteins than by a complete substitution of COLIV by COLI, we assessed EPO synthesis when cells were plated on a collagen mixture with variable COLI and COLIV concentrations, and we observed differences in EPO synthesis depending on the ratio COLI/COLIV concentrations in the collagen mixture. Thus, our results support the biological relevance of the COLI‐dependent EPO downregulation as these results show a relationship between ECM composition and EPO synthesis. To our knowledge, this is the first report of the relationship between composition of collagen types and EPO synthesis in renal cells. However, it has been suggested that renal fibrosis may determine phenotypic changes in interstitial fibroblasts in response to microenvironmental changes that would range from an EPO synthesizing to a matrix producing phenotype, thus conditioning a decreased EPO synthesis with the subsequent anaemia 41.

HIF is the main responsible transcription factor for the EPO induction 24. Although some papers have reported that HIF can be regulated by integrins in hypoxic conditions 52, there is no clear evidence of this regulation. As a number of evidence show that isoform HIF2α can be the main regulator of EPO *in vivo* in adult kidney (26, 32), we decided to study the regulation of this isoform. We observed that HIF2α protein and activity were down‐regulated in cells cultivated on COLI with respect to COLIV, without changes in mRNA expression, suggesting a non‐transcriptional regulation but an increased HIF2α degradation. To determine the mechanism through which HIF2α is excessively degraded in TK173 cells plated on COLI, we studied some proteins involved in HIF pathway such as PHD and VHL. We analysed PHD isoforms, but only PHD2 is expressed in these cells. We found that PHD2 mRNA, protein expression and its activity measured as HIF hydroxylation were similar in cells on COLI with respect to COLIV. Moreover, VHL mRNA and protein expression were not modified by the type of collagen (data not shown). As HIF2α is degraded by proteasome, we measured proteasome activity and observed that it was significantly higher in cells harvested on COLI than in those on COLIV. When we inhibited proteasome activity with the inhibitor MG132, EPO mRNA levels and HIF2α protein expression in cells on COLI were similar to those observed in cells on COLIV. Although it has been described that proteasome inhibitors do not increase HIF activity but only the amount of protein, in our hands it increases not only cellular HIF2α protein content but also restore interstitial fibroblast EPO synthesis to a level similar to that observed for COL IV. Additional studies are needed to better clarify these effects, but our results propose an alternative way to modulate HIF2α protein levels, and subsequently EPO synthesis, in CKD patients.

FAK, and not ILK, seems to be the protein responsible for COLI‐induced changes in EPO synthesis because neither ILK protein expression nor activity, measured as GSK3ß phosphorylation, changed in cells cultured on COLI compared with those on COLIV. In contrast, FAK phosphorylation at Tyr^397^ was significantly higher in cells on COLI than in those on COLIV, without changes in total‐FAK content, supporting a higher activation of the protein after exposition to COLI. Moreover, FAK blockade by mRNA depletion or pharmacological treatment inhibited the COLI‐dependent increased proteasome activity and restored the decreased HIF2α protein content and EPO mRNA expression to values similar to those observed in fibroblasts cultured on COLIV. This finding constitutes an additional support to the biological relevance of the interaction between fibroblasts and ECM in the process of EPO synthesis, as FAK is considered a critical protein in the regulation of the cross‐talk ECM cells.

To gain more insight into the pathophysiological meaning of our results in cells, as well as to explore new possibilities of increasing EPO synthesis in chronically damaged kidneys, we used a model of tubulointerstitial fibrosis induced by UUO. This model is characterized by the presence of myofibroblast from different origins and increased accumulation of ECM proteins at interstitial level 53, 54. In the obstructed kidney of UUO mice, it was observed tubulointerstitial fibrosis, a significant increase in COLI and a significant decrease in EPO mRNA expression, with respect to the contralateral non‐obstructed kidneys. These mice did not have anaemia because the ureteral obstruction was performed only in one kidney.

Although FAK phosphorylation was higher in the obstructed than in non‐obstructed kidneys, the differences were not statistically significant. Protein phosphorylation is a rather quick mechanism of regulation of their activity, and it may occur that after 15 days of obstruction the changes in FAK phosphorylation were no longer detected. On the other hand, we did not observe the reduction of HIF2α content that we observed in cells grown on COLI, in the fibrotic kidneys. Surprisingly, we observed a marked increment of the level of this protein in total renal cortex (data not shown), apparently in contradiction with the almost complete disappearance of EPO expression. It should be noted that total cortex analysed in the kidneys is composed with many cell types, probably with different behaviour with respect to the phenomenon studied, and the most predominant cell type are tubular epithelial cells. To analyse this discrepancy, we performed an immunohistochemistry to study HIF2α expression. The protein increased in tubular epithelial cells, but not in EPO‐producing cells, interstitial fibroblast, in which EPO was completely absent. Interestingly, obstructed kidneys of mice treated with the proteasome inhibitor bortezomib showed a level of EPO synthesis similar to non‐obstructed kidney levels. These data support our *in vitro* findings, suggesting that proteasome activity is augmented in fibrosis, and it could be involved in EPO downregulation observed.

In summary, our data point to a new mechanism of anaemia development in patients with CKD. The *in vitro* findings clearly support that COLI accumulation that takes place in renal fibrosis induces FAK activation in EPO‐producing interstitial fibroblasts, which in its turn increases proteasome activity. Proteasome degrades HIF2α with the subsequent EPO downregulation. *In vivo* findings also support this contention.

## Author contribution

GO, JMM‐F and IM performed experiments. GO and DR‐P were involved in the conception and design of research. GO, MPR‐T, JMM‐F, JML‐N and DR‐P analysed data, interpreted results and prepared figures. GO, MPR‐T and DR‐P edited and revised manuscript. GO, JM M‐F, IM, MPR‐T, AGM, JML‐N and DR‐P approved final version of this manuscript. All authors were involved in the critical and review of manuscript.

## Conflict of interest

The authors confirm that there are no conflict of interests.
